# Charity Lotteries

**Published:** 1890-01-11

**Authors:** 


### Fallow Crops.

### CHARITY LOTTERIES.

We cannot affect to be either profoundly surprised or greatly regretful that the proposed lottery scheme in connection with the North-West London Hospital has been interdicted. Of course, we sympathise with the charity, in all respects a most estimable one, but anything like indignation appears entirely out of place, for when once the attention of the authorities was directed to the proceedings there could be no alternative to the course pursued.

Doubtless the hospital is unfortunate, as things not altogether unlike that now disapproved of have been done with impunity elsewhere, and if, as is averred, a Roman Catholic church in the same neighbourhood has been allowed recently to raise money in a precisely similar fashion, an explanation seems desirable. But this would be in order to find out why the Roman Catholics were permitted to proceed rather than why the North-West London Hospital has been pulled up. For unless it is to be understood that in the cases of churches and charities the law is to be a dead letter, where can be the excuse for its open violation, whether by the church or the hospital? The managers of the latter have been caught *in flagrante delicto*, and learn, as many other well-intentioned culprits before them, that in the legal code of ethics, at any rate, the end is not held to justify the means.

Regard this matter from what point of view we may, one fact stands out clearly—the proposal for a "presentation of prizes" was a prospectus of a lottery pure and simple, in which the first prize was of the value of £200, receivable, if the winner so willed, in hard cash. This offer of money in place of the picture may be taken to have removed any particle of doubt as to the illegality of the proceedings, which otherwise might have been entertained.

The contention put forward amiably by a contemporary that "the class of buyers of the tickets is the least likely of all to be influenced by considerations of profit" will not bear examination. It was the hope of possible profit which was held out as an inducement, and if the scheme was to be successful it would have been so for no other reason than because the lure proved attractive. The record of our hospitals only too plainly, too cruelly, demonstrates that the satisfaction of participating in the support of a good work is no inducement at all to the public in general, and that some even of the regular subscribers are by no means averse from demanding a very considerable *quid pro quo* in the way of patronage or otherwise.

Obviously, the object held in view by the promoters of this, as of other similar efforts, was to obtain contributions from people outside the limited class who give from motives of pure philanthropy, and it is easy to conceive regrettable and even scandalous incidents as likely to arise in connection with a private lottery, where the chance of a valuable cash prize is secured by anyone who cares to risk half-a-sovereign.

Is it not to be deplored that institutions of acknowledged utility should be left to depend upon such precarious means of support that their managers must be for ever cudgelling their brains to find fresh methods of raising funds? One cannot but sigh over the loss of that spirit of true piety which in the earlier periods of the Christian era linked religion to all works of practical benevolence—to such good purpose that the offerings to Charity were brought by willing hands and laid reverently at her feet. Now she, who ought to be sacred, is forced to descend to the street to ply the pitiful part of beggar at every corner and stoop to any act which "doth give her bold advertisement."

If we regret that a worthy hospital has been disappointed of much-needed assistance, we cannot avoid the reflection that the extent to which it is a loser by the interposition of the Home Secretary is the exact measure of the result sought to be obtained by a method unorthodox and, in a sense, unbecoming. Agricola.

